# Cancer in Angola, resources and strategy for its control

**Published:** 2012-05-23

**Authors:** Lygia Vieira Lopes, Ana Vaz Conceição, João Blasques Oliveira, António Tavares, Clarinha Domingos, Lucio Lara Santos

**Affiliations:** 1 Sagrada Esperança Clinic, Angola; 2 Girassol Clinic, Angola; 3 Doctors of the world, Portugal; 4 Angolan National Centre of Oncology, Angola; 5 Military Hospital, Portugal; 6 Portuguese Institute of Oncology, Portugal

**Keywords:** Angola, cancer care, resources, strategy

## Abstract

**Background:**

Cancer is an increasingly important health problem in Africa. The number of cancer cases in this region could double, ranging between 700 000 and 1 600 000 new cases in 2030. The mortality rate is higher than 80% and is explained, mainly, by a lack of early detection, diagnostics and treatment resources. In Angola, about 7,000 patients die of cancer every year.

**Methods:**

Data were derived from open-ended interviews conducted in 2010-11 with health authorities, clinicians, nurses and Administration of Hospitals. According Angola epidemiological data, results of interviews and international published advocacy for cancer control we develop a potential strategy for its control. The objectives are to identify existing resources for cancer control and describe the needs thereto, in order to establish an oncological program to guide the development of Angola cancer control strategies.

**Results:**

Malaria remains the leading cause of illness and death in Angola, and other communicable diseases remain a public health problem. However, 9 000 new cases of cancer are diagnosed each year.The most common types of cancer are: cancer of the cervix, breast, prostate, esophagus, stomach and head and neck, as well as cancers with infectious origin, such as Kaposi‘s sarcoma and liver and bladder cancer. The foundation for developing national cancer control strategies includes: oncological data; investment and training; identifying and removing barriers; guidance and protection of the patient. Angolan National Cancer Centre, Sagrada Esperança Clinic and Girassol Clinic are now developing a cancer program.

**Conclusion:**

Improving the economic situation of Angola creates conditions for an increase in life expectancy which in itself is associated with an increased risk of oncological diseases. On the other hand, infectious diseases, associated with the risk of malignant tumors, are endemic. Thus, an increase in patients with malignant disease is expected. A plan is therefore necessary to organize the response to this old but less visible nosologic situation.

## Background

Low and middle income countries, that constitute the majority of African nations, have witnessed a 20% reduction in the per-capita global burden of disease (GBD) since 1990, due to a decrease in infectious/communicable diseases, malnutrition and neonatal illnesses [1].

By contrast, in those same countries non-communicable diseases are rising and are now responsible for 50% of the GBD. In this group of non-communicable diseases, the most relevant in prevalence are cerebrovascular and malignant diseases [2].

Africa has a complex demographic and epidemiological situation that defines the context of any health intervention: it has a very young population - 41% of which is under 15 years old-associated with a high fertility rate of 4.7 births per woman, but a growing number of Africans are now living beyond the age of 50 years. The UN estimates that people aged 60 years or more will make up almost 26% of the African population by 2050 [3]. In adults over 45 years old, the cause of death in 62% of the cases is related to non-communicable diseases, while only 33% are related to communicable diseases.

Agyei-Mensah showed that the high rate of urbanization associated with very important discrepancies in wealth and increasing urban poverty numbers were associated with a double burden of diseases and epidemiological models. These include huge levels of infectious diseases as the main cause of illness and death, as well as an increasing number of chronic and non-communicable diseases, also leading to death and significant disability, and caused first by cardiovascular diseases but also by diabetes and cancer [4].

Therefore, cancer is an increasingly important health problem in Africa. According to Dr. Luis Sambo, the WHO Regional Director for Africa, the number of cancer cases in the region could double, ranging between 700 000 and 1 600 000 new cases in 2030. The mortality rate is higher than 80% and is mainly explained by a lack of early detection, diagnostic and treatment resources [5]. The most common types of cancer are: cancer of the cervix, breast, prostate, oesophagus, stomach, head and neck, and cancers with a infectious origin, such as Kaposi‘s sarcoma and liver and bladder cancers.

Oncologic diseases in Angola are still of minor importance, compared to infectious diseases, but their mortality rate is high. According to Globocan (2008) for Angola, cancer of the cervix is the most common, breast cancer being the second most common in females. In males, prostate cancer is the most prevalent, followed by hepatocellular carcinoma. Malignant tumors associated with HIV and with schistosomiasis are also relatively common [6, 7].

The aim of this study is to identify existing resources for cancer control and describe the needs thereto, in order to establish an oncologic program to guide the development of cancer control strategies in Angola.

## Methods

The data, including information about nosologic issues and specific resources, were obtained from open-ended interviews to health authorities, clinicians, nurses and hospital administrations conducted in 2010 and 2011. A literature review specifically focused on “cancer in Angola” was performed. Citation indexes were searched in Embase and PubMed databases. Taking into account the epidemiological data published for Angola, associated with the results of the interviews and with the international rules published and advocated for cancer control in low and middle income countries, we were able to achieve sufficient knowledge to draw up a potential strategy for cancer control in Angola.

## Results

Angola has just over 2200 doctors for about 16 million people with a life expectancy at birth of 47 years. Malaria remains the leading cause of illness and death, but its mortality rate is decreasing. HIV is another public health problem. The seroprevalence is 1.9% and about 20 new cases of HIV are detected every day. The Angolan State provides about 35 million dollars a year to combat this disease. Angola has five medical schools and there are several professional schools for nurses and other health professionals [8, 9].

In Angola there are about 9000 new cases of cancer diagnosed each year, and there are 7000 deaths attributed yearly to this disease (Table 1). In 2006, 621 new cases of cancer were admitted to the National Oncology Center in Luanda. Two thirds were females and of these female cases 6% were under 15 years old. Twenty-four percent of these tumors were located in the breast, 16% in the uterine cervix, 9% were hematological, 7% were Kaposi‘s sarcomas, 5% were skin, non-melanoma, and 3% were hepatocellular carcinomas. Fifty-three percent were in an advanced stage at the time of diagnosis [6, 7, 10].

**Table 1:** Angola - Most frequent cancers: male and female according (Globocan 2008)

Cancer	Males				Females			
	Incidence		Mortality		Incidence		Mortality	
	Number	ASR (W)	Number	ASR (W)	Number	ASR (W)	Number	ASR (W)
Lip, Oral cavity	195	5.0	88	2.6	118	2.6	54	1.3
Nasopharynx	38	0.8	24	0.6	23	0.4	15	0.3
Other pharynx	64	1.8	54	1.6	16	0.4	13	0.3
Oesophagus	188	5.2	180	5.0	111	2.6	107	2.6
Stomach	195	4.6	184	4.5	195	3.8	186	3.7
Colorectum	191	4.5	150	3.7	152	3.2	117	2.6
Liver	552	10.9	539	11.1	331	6.0	325	6.6
Gallbladder	1	0.0	1	0.0	10	0.2	10	0.2
Pancreas	42	1.1	42	1.1	66	1.3	62	1.3
Larynx	116	3.4	70	2.2	13	0.3	8	0.2
Lung	126	3.3	118	3.2	56	1.4	52	1.3
Melanoma skin	84	2.0	52	1.3	66	1.4	38	0.9
Kaposi sarcoma	186	3.7	161	3.3	32	0.5	25	0.4
Prostate	611	19.7	463	16.3	-	-	-	-
Breast	-	-	-	-	1004	19.5	558	11.9
Cervix uteri	-	-	-	-	1504	30.0	1008	21.9
Corpus uteri	-	-	-	-	95	2.1	32	0.8
Ovary	-	-	-	-	187	3.5	140	3.0
Testis	20	0.3	12	0.2	-	-	-	-
Kidney	81	1.4	68	1.1	77	1.1	67	0.9
Bladder	92	2.6	65	2.1	37	0.9	28	0.7
Brain NS	22	0.4	21	0.4	17	0.3	17	0.3
Thyroid	29	0.6	19	0.5	64	1.2	37	0.9
Hodgkin L	39	0.7	33	0.6	27	0.4	23	0.4
Non-Hodgkin L	238	4.0	199	3.3	223	3.5	186	2.9
Multiple Myeloma	34	0.9	31	0.9	28	0.7	25	0.6
Leukaemia	134	2.2	127	2.1	99	1.5	93	1.4
All cancers	3991	92.9	3338	80.2	5207	99.7	3723	76.3

### Angolan Resources

The National Oncology Center in Luanda is the oldest public center for the treatment of cancer patients in Angola. It has doctors, nurses, technicians and diagnostic capabilities. It also has chemotherapy facilities and professionals with experience in chemotherapy. There is a Varian^®^ radiotherapy equipment, but it is not running yet. A network for data collection inside the country exists and sometimes acts as a referral service [10].

**Figure 1:**
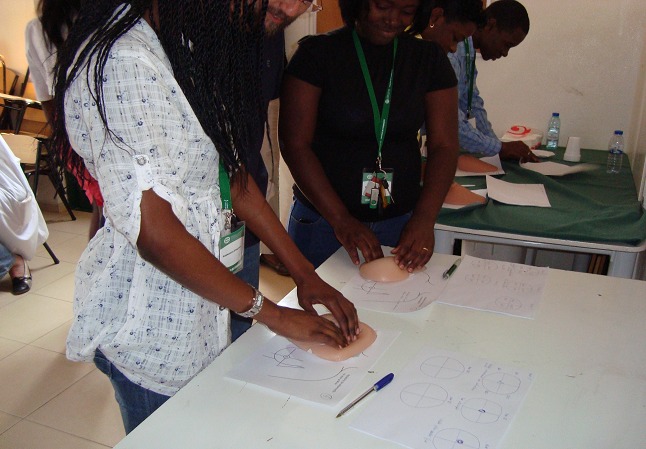
Breast cancer family doctors training, Sagrada Esperança Clinic

**Figure 2:**
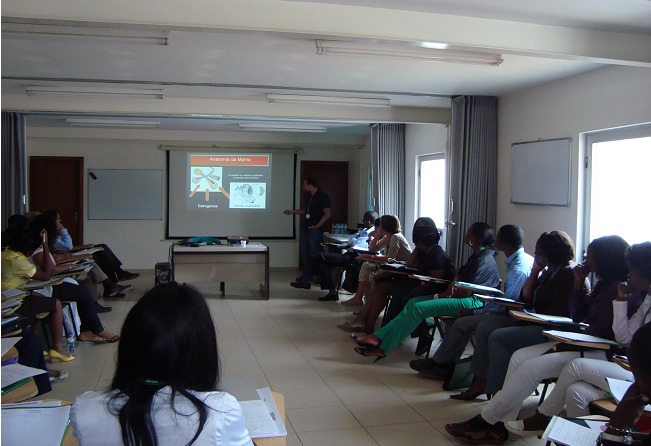
Medical post-graduation course in Oncology - Luanda

The Sagrada Esperança Clinic belongs to a public company named Endiama. In addition to imaging and laboratory facilities, this clinic also has pathology facilities and a unit for the diagnosis, treatment and monitoring of breast pathology, with multidisciplinary treatment decisions implemented. There are two training programs currently in progress: breast oncology for general practitioners and nurses, and a training program for urologists (Figure 1 and Figure 2). This clinic is also experienced in the surgical treatment of oncology, including breast. It is presently organizing a chemotherapy service, as well as a unit for control of chronic pain and a breast cancer screening program for Endiama employees.

The Girassol Clinic belongs to the public company Sonangol. This clinic has the capacity for diagnostic imaging, as well as laboratory facilities. It also has a specifically built oncology service, with a dedicated ward, a day care hospital, a radiotherapy unit (two sets of Artiste equipment of Siemens^®^), and Nuclear Medicine resources for which operational licenses are expected to be issued by the Atomic Energy Agency. This clinic has now started organizing this oncologic service and the first steps for cancer care have been taken.

The Américo Boavida Hospital, the David Bernardino Pediatric Hospital and the Josina Machel Hospital, are all teaching hospitals where most of the cancer patients are diagnosed. In these hospitals, there are diagnostic capabilities and experience in the surgical treatment of some cancers. Elsewhere in Angola, the provincial hospitals are the main places of diagnosis, referring patients to Luanda.

### Partnerships

The Sagrada Esperança Clinic and the Girassol Clinic have the support of the Portuguese Institute of Oncology in Porto, Portugal, which is a comprehensive cancer center with OECI (Organization of European Cancer Institutes) accreditation [11].

## Discussion

Cancer is already one of the leading causes of death in developing countries, including in Angola. The incidence of cancer in developing countries is growing because of improvements in the control of communicable diseases and the resulting increase in life expectancy [12]. Only 5% of global resources for cancer control are currently spent in developing countries. Up to one third of new cancer cases in developing countries could be prevented with proper awareness programs and other implementable primary prevention measures. Survival rates for one third of the patients could be increased if the cancer were detected earlier [12].

On the other hand, facilities for the diagnosis and treatment of cancer in most developing countries are scarce. Thus, the primary objective is the construction of a coherent program in a relatively short time, in which health gains are observed in a consistent manner, without forgetting the fundamental measures of primary and secondary prevention. This program will consist of organizational aspects and staff training, and will require some investment.

The strategy to combat the malignancy involves: investment and training; identification and removal of barriers; and guidance and protection of the patient, as described by Bridges et al. in the control of breast cancer [13].

### Oncologic Program

Any non-communicable disease control program, including cancer control, must be built, from the beginning, on a comprehensive strategy that should include prevention, promotion of healthy behaviors, adequate and timely diagnosis and corresponding multidisciplinary treatment. This can be achieved by addressing the weaknesses of the health systems as a whole, particularly those of the existing hospital system at all levels [14].

The lack of appropriate resources for biopsies and limited training of doctors in the early detection of cancer are some of the constraints that impair the ability to have a clear picture of the epidemiology of cancer in Angola, and must be solved within the health system.

The foundation of a cancer program includes the following tasks: organization, training, investment, health education and behavioral change.

### Organization

Keeping a Cancer Registry is a very important activity, because it allows assessment of the magnitude of the problem and is instrumental for the National Oncology Program in setting priorities and determining the resources which need to be allocated. The identification of the existing material and human resources also provide strategic information. The integration and cooperation between health facilities dedicated to the diagnosis and treatment of cancer is crucial to increase proficiency in this area, avoiding unnecessary duplications [15].

### Training

The training of dedicated medical staff should be associated with the acquisition of proper equipment [16]. A multidisciplinary decision model for establishing treatment and auditioning care quality and clinical results requires learning.

### Investment

Investment in diagnostic facilities, pathology, surgical capacities, chemotherapy, radiotherapy and palliative care resources is fundamental and necessary [17, 18].

### Health education and behavioral change

Cancer control must rely on health promoting activities and preventive interventions. It is known that at least in some cancers, behaviors such as smoking and heavy drinking are related to a higher risk of malignant transformation. It is also known that the best results in cancer treatment are achieved with early detection, and this notion should be transmitted and promoted among the population. Thus, breast and oral cavity self-examination, as well as cervical cytology, should be encouraged. The urologic observation should also be stimulated in case of specific symptoms or age related risks [19].

### Appropriate protocols

As is advocated by Gueye SM, it is simply unethical to screen the population and not have the capabilities to offer treatment for the detected diseases [20]. The occupational health services are present in most Angolan and foreign companies working in Angola. These services should have an important role in early diagnosis and prevention of most common malignancies. The therapeutic recommendation protocols in the area of oncology should take into account the resources available, the stage and biologic characteristics of the disease, the life expectancy of the population and the profile of the country, as well as the statements of the Global Task Force on Expanded Access to Cancer Care and Control in Developing Countries or the Breast Health Global Initiative [18].

## Conclusion

The improving economic situation of Angola creates conditions for an increase in life expectancy, which in itself is associated with an increased risk of oncologic diseases. On the other hand, infectious diseases associated with the risk of malignant tumors are endemic. Thus, an increase in patients with malignant diseases is expected. It is therefore necessary to organize the response to this less visible nosologic situation.
